# COVID‐19 risk perception and associated factors in older adults in southern Ethiopia

**DOI:** 10.1111/irv.13042

**Published:** 2022-08-28

**Authors:** Tadese Dea Debancho, Eyasu Gambura Gebeyehu, Temesgen Bati Gelgelu

**Affiliations:** ^1^ Boloso Sore Woreda Health Office Wolaita Zone Health Department Wolaita Sodo Ethiopia; ^2^ School of Public Health, College of Health Sciences and Medicine Wolaita Sodo University Wolaita Sodo Ethiopia

**Keywords:** coronavirus, Ethiopia, older adults, risk perception

## Abstract

**Background:**

COVID‐19 remains a public health concern in lower income countries. Risk perception has been studied in different countries with different population groups. However, there have been few studies conducted risk perception on older adults and limited data from African continent. This study aimed to assess coronavirus disease low risk perception level and associated factors among older adults in Ethiopia.

**Methods:**

We conducted a cross‐sectional study among older adults in Areka town, Wolaita Zone, Southern Ethiopia, from August 1, 2021, to August 30, 2021. Multi‐stage sampling method was applied to select study participants. The data were collected through a structured questionnaire with the mobile application created with Open Data Kit mobile.

**Results:**

Overall, risk perception was fairly low. Risk perception was particularly low among individuals aged 65 to 74 years (AOR = 4.76, 95% CI: 2.35–9.64), poor practice on preventing coronavirus disease (AOR = 2.39, 95% CI: 1.51–3.78), with low trust level in medical professionals (AOR = 2.44, 95% CI: 1.45–4.10), no history of coronavirus disease (AOR = 6.45, 95%CI [2.02–20.58]), and poor perceived self‐efficacy for preventive practice (AOR = 2.25, 95% CI: 1.43–3.54).

**Conclusions:**

In the current study area, the perception of risk of coronavirus disease was affected by age, perceived self‐efficacy, trust in medical professionals, preventive practice, and history of COVID‐19. The findings of this study would help lower income countries to generate evidence‐based policy decisions for older adults during the COVI‐D‐19 pandemic and future pandemic(s).

## 1 INTRODUCTION

Coronavirus disease 2019 (COVID‐19) was first detected in China in December 2019 in Hubei Province and is caused by the severe acute respiratory syndrome corona virus 2 (SARS‐CoV‐2). The disease was officially declared as a public health emergency of international concern on January 30, 2020, and a global pandemic on March 11, 2020.^1^ The continued evolution of new variants has been a major challenge for the success of combating coronavirus pandemics in the world. COVID‐19 remains a public health concern in both higher income and lower income countries.^2^ As of April 2022, more than 500 million confirmed cases with more than 6 million deaths have been reported globally, including over 8.5 million confirmed cases and 171,457 deaths in Africa, and 470,352 cases and 7,510 deaths in Ethiopia.^3^

The pandemic has caused great fear, and the group that has suffered the most are older adults across the world.^4^ Because of physiological changes due to ageing as well as a generally higher incidence of underlying medical conditions, older adults face the highest risk of developing severe illness if infected.^5^

Understanding how people are being presented with risky situations during the COVID‐19 pandemic, how people are assessing these risks, and how such assessments lead them to change their behaviors are essential to inform COVID‐19 health recommendations.^2^ Risk perception refers to people's attitudes, beliefs, judgments, and feelings toward risk.^6^ People with high risk perception are more likely to practice preventive behaviors and vice versa.^7^Additionally, multiple health models suggest that COVID‐19 risk perception level is an important component for changing the health behaviors of the community.^8^ Risk perception of an individual is influenced by different factors.^9^ Risk perception has been studied in different countries with different population groups. However, there have been few studies which assessed risk perception in older adults and there is no study on COVID‐19 risk perception level in older adults in Ethiopia. Thus, gathering basic information about the vulnerable group and assessing how they perceive COVID‐19 is the first step in involving risk communication.^10^

## 2 MATERIALS AND METHODS

### 2.1 Study design, period, and setting

To carry out this study among older adults in Areka town, Wolaita Zone, SNNPR, from August 1, 2021, to August 30, 2021, a community‐based cross‐sectional study design was used. It is an administrative center of Boloso Sore Woreda. It is located 300 km far from the capital city of Ethiopia, Addis Ababa, in south‐western direction. Based on population estimation in 2020, it has an estimated total population of 67,000, of whom 33,000 are men and 34,000 are women. The total number of adults ≥65 years of age in Areka town is 2386.

### 2.2 Source and study population

The source population was all older adults (≥65 years) in Areka town, Wolaita Zone. The study sample comprised randomly selected older adults (≥ 65 years) community members in the selected subtown administration of Areka town and fulfilled the inclusion criteria.

### 2.3 Sample size determination and sampling procedure

To determine the sample size, we used a single population proportion formula with the following statistical assumptions: *p* = 50% (there was no study on the level of risk perception which was done on older adults), *d* (margin of error = 5%), *Z*_α/2_ (standard score value for 95% confidence level = 1.96), and design effect (1.5). After using the correction population formula and adding 10% non‐response rate, the final sample size was 547. To select an individual's household, we applied a multistage sampling technique. At the first stage, the subtown administration was selected randomly, and then, at the second stage, villages existing within subtown administration were randomly selected. Finally, the individual household was selected using systematic sampling techniques. The study subjects for the study in each village were selected by using systematic randomly sampling every third household from the sampling frame compiled in each village. The first household was selected randomly by lottery method from the first three households.

### 2.4 Data collection instrument

The mobile application of Open Data Kit was used to collect the data. Every day, the collected data was transferred to the server and the assigned supervisor in each subtown followed the operation. Seven nurses who had previous experience with the mobile applications were participated in data collection. The data collection instrument was executed and developed by reviewing different literatures.^11^, ^12^, ^13^, ^14^, ^15^

### 2.5 Data quality

The instrument was first prepared in English and then translated to Amharic and Local (Wolaitic) languages, and then after, it was translated back to English language to check its consistency. The data were collected by trained individuals. Pretest was conducted upon 5% of older adults outside the study area in Mehal subtown administration unit.

### 2.6 Data processing and data analysis

Comma Separated Value (CSV) format was used to export data by using mobile application Open Data Kit Briefcase‐v1.17.4 software and imported to SPSS version 20 for analysis. Descriptive summary was reported by using frequency and percentage. For data analysis, logistic regression was applied. Multi‐collinearity of the independent variables was checked by the diagnostic test of variance of inflation factor (VIF < 10) prior to the running of logistic regression. Bivariate logistic regression analysis was carried out to distinguish the association between independent and dependent variables. Then, *P* < 0.20 variables were candidates for multivariable logistic regression. Statistical significance was set at *P* < 0.05. The final logistic model of the goodness of fit was tested using Hosmer and Lemeshow test at a 𝑝 value of >0.05.

### 2.7 Variable measurement

COVID‐19 risk perception level was measured by 12 items which responses were rated on Likert scales as *strongly disagree* (1), *disagree* (2), *neutral* (3), *agree* (4), and *strongly agree* (5). Cumulative score of risk perception was computed which ranges from (lowest score [12] to highest score [60]), and the mean score was used to categorize risk perception in to “high risk perception” and “low risk perception.” Those of respondents having higher value than the mean score were categorized as high risk perception and below, or the mean value was categorized as low risk perception.^12^, ^13^, ^14^, ^15^

The practice of COVID‐19 prevention method was measured using nine questions adapted from previous research. Responses were rated as *none* (1), *rarely* (2), *sometimes* (3), *frequently* (4), and *always* (5). Finally, the cumulative practice score was computed, which ranged from 9 to 45. Those of individuals who were correctly answered with a mean value or less than the mean value were classified under having poorly practiced the prevention method of COVID‐19, and above mean value was classified as having practiced well toward the prevention methods of COVID‐19.^12^

Knowledge‐related factors of COVID‐19 were measured using 15 items. The questions were answered on *yes*, *no*, or *I do not know* responses. During analysis, the correct answer was coded as “1,” and incorrect answer or an *I do not know* answer was coded as “0.” Then, the sum was calculated, ranging from 0 to 15, and categorized based on the cumulative score. Participants who answered >75% of the items correctly were classified as having good knowledge, those who answered 61–75% had fair knowledge, and those who answered ≤60% of the questions correctly were considered as having poor knowledge.^12^

## 3 RESULTS

### 3.1 Socio‐demographic characteristics of participants

A total of 537 respondents were enrolled in the study, corresponding to a 98.2% response rate. The mean age of the participants was 75. Above half 287 (53.4%) of the participants were found in the age group of between 65 and 74 years. About 278 (51.8%) of the respondents were males. Three fifth of participants, 327 (60.9%) were married. About 172 (32.0%) can read and write. About 204 (38.0%) were employed, and three quarters 411 (76.5%) have family monthly income of greater than 2000 ETB (Table 1).

**TABLE 1:** Socio‐demographic characteristics of the participants in Southern Ethiopia, 2021 (*n* = 537)

Variables	Categories	Frequency	Percent
Age	65–74	287	53.4
	75–84	199	37.1
	85 and above	51	9.5
Sex	Male	278	51.8
	Female	259	48.2
Marital status	Married	327	60.9
	Single	14	2.6
	Divorced or separated	43	8.0
	Widowed	153	28.5
Educational status	Cannot read and write	91	16.9
	Read and write	172	32.0
	Grade 1–8	37	6.9
	Grade 9–10	23	4.3
	Grade 11–12	33	6.1
	Diploma and above	181	33.8
Occupation	Employed	204	38.0
	Merchant	170	31.7
	Housewife	79	14.7
	Farmer	42	7.8
	Daily laborer	42	7.8
Family monthly income	<750	25	4.7
	750–1,300	16	3.0
	1,301–2,000	85	15.8
	>2,000	411	76.5

### 3.2 COVID ‐19 risk perception of respondents

About one quarter, 146 (27.6%) and 143 (26.6%) of the participants strongly agree on the perception that getting sick with coronavirus can be serious and their health will be severely damaged if they get sick with coronavirus disease, respectively. One‐hundred fifty nine (29.6%) disagreed with the perception that recovering was not possible if someone was infected by COVID‐19. Above half 309 (57.5%) of participants agreed with the perception that COVID‐19 was causing more deaths than other respiratory conditions. Half 269 (50.1%) agree with the perception that people can be stigmatized if they get sick due to COVID‐19. About 286 (53.3%) disagree with the perception that people can contract COVID‐19 even if they take preventive measures. About three fifth, 331(61.6%) of participants disagree with the perception that they contract COVID‐19 even if they do not come in to contact with a COVID‐19 patient. The proportion of low risk perception level of COVID ‐19 among older adults was 51.8%, 95% CI (47.5%, 56.0%). The mean score value of risk perception level of COVID‐19 was 41 (Table 2).

**TABLE 2:** Risk perception level on CIVID‐19 among older adults participants in Southern Ethiopia, 2021 (*n* = 537)

Items	Strongly disagree *N* (%)	Disagree *N* (%)	Neutral *N* (%)	Agree *N* (%)	Strongly agree *N* (%)
Getting sick with the coronavirus can be serious	0 (0.0%)	0 (0.0%)	15 (2.8%)	376 (70.0%)	146 (27.2%)
My health will be severely damaged if I contract coronavirus	0 (0.0%)	4 (0.8%)	48 (8.9%)	342 (63.7%)	143 (26.6%)
It is not possible to recover from coronavirus disease	28 (5.3%)	159 (29.6%)	78 (14.5%)	152 (28.3%)	120 (22.3%)
Coronavirus causes deaths more than other respiratory diseases	2 (0.4%)	15 (2.8%)	74 (13.8%)	309 (57.5%)	137 (25.5%)
If I caught with coronavirus, I cannot manage my daily activities	1 (0.2%)	27 (5.1%)	98 (18.2%)	297 (55.3%)	114 (21.2%)
People may stigmatize me if get due to coronavirus	1 (0.2%)	31 (5.8%)	133 (24.8%)	269 (50.0%)	103 (19.2%)
I think that I will contract coronavirus if you do not take any preventive measures	41 (7.6%)	283 (52.7%)	113 (21.1%)	69 (12.8%)	31 (5.8%)
I think that I will contract coronavirus if you take preventive measures	54 (10.1%)	286 (53.3%)	127 (23.6%)	64 (11.9%)	6 (1.1%)
I think that I will contract coronavirus if I come into contact with a coronavirus patient	38 (7.1%)	106(19.7%)	96 (17.9%)	113(21.0%)	184(34.3%)
I think that I will contract coronavirus even if I do not come into contact with a coronavirus patient	7 (1.3%)	42 (7.8%)	134 (25.0%)	270 (50.3%)	84 (15.6%)
The coronavirus will not affect very many people in the area I am currently living	28 (5.2%)	109 (20.3%)	96 (17.9%)	121 (22.5%)	183 (34.1%)
My work exposes me more to coronavirus than another person	106 (19.7%)	124 (23.1%)	83 (15.5%)	101 (18.8%)	123 (22.9%)

### 3.3 Knowledge regarding COVID‐19

Nearly three quarters (72.4%) of the older adults had good knowledge, which is followed by fair knowledge (17.9%) and poor knowledge (9.7%) toward COVID‐19. About 527 (98.1%) respondents knew about coronavirus disease. About 508 (94.6%), 480 (89.4%), 519 (96.6%), 477 (88.8%), 87 (16.2%), and 15 (2.8%) thought that the COVID‐19 can be transmitted through a droplet from an infected person, air‐borne, direct‐contact, contact with contaminated objects, and contact with contaminated animals and mosquito bites, respectively. About 505 (94.0%), 527 (98.1%), and 508 (94.6%) knew that COVID‐19 had symptoms experiencing fever, cough, and shortness of breath, respectively (Table 3).

**TABLE 3:** Items used to assess knowledge of COVID‐19 among older adults in Southern Ethiopia, 2021 (*n* = 537)

Items		Yes	No	I do not know
Do you know that the new coronavirus/COVID‐19 disease was one of respiratory disease?		527 (98.1%)	8 (1.5%)	2 (0.4%)
Coronavirus disease would be transmitted through	Droplet from infected people	508 (94.6%)	22 (4.1%)	7 (1.3%)
	Airborne	480 (89.4%)	29 (5.4%)	28 (5.2%)
	Direct contact with infected people	519 (96.6%)	12 (2.2%)	6 (1.1%)
	Touching contaminated object/surfaces	477 (88.8%)	37 (6.9%)	23 (4.3%)
	Contact with contaminated animals	87 (16.2%)	383 (71.3%)	67 (12.5%)
	Mosquito bites	15 (2.8%)	380 (70.8%)	142 (26.4%)
The individual contracted by coronavirus disease would experience	Fever	505 (94.0%)	20 (3.7%)	12 (2.2%)
	Cough	527 (98.1%)	8 (1.5%)	2 (0.4%)
	Shortness of breath/breathing difficulties	508 (94.6%)	12 (2.2%)	17 (3.2%)
Coronavirus disease would be prevented though mechanisms	Wash your hands regularly using alcohol or soap and water	445 (82.9%)	89 (16.6%)	3 (0.6%)
	No treatment or vaccine for the COVID 19.	406 (75.6%)	97 (18.1%)	34 (6.3%)
	Avoid close contact with anyone who has a fever and cough	447 (83.2%)	89 (16.6%)	1 (0.2%)
	Avoid unprotected direct contact with live animals and surfaces	118 (22.0%)	413 (76.9%)	6 (1.1%)
	Sleep under the mosquito net	9 (1.7%)	513 (95.5%)	15 (2.8%)

### 3.4 Preventive practice toward COVID‐19

About 288 (53.6%) had poor practice of prevention methods against COVID‐19. About 99 (18.4%) of the participants frequently practiced maintaining physical distance from others. One‐hundred one (33.7%), 207 (38.5%), and 259 (48.2%) rarely practiced avoiding of larger gathering, contacting with people who had fever and cough, and frequently touched surfaces, respectively. Two fifths, 204 (38.0%) of participants rarely practiced washing of hands regularly with soap and water/sanitizer. The mean score of preventive practice on COVID‐19 was 24 (Table 4).

**TABLE 4:** Preventive practice toward COVID‐19 among older adults in Southern Ethiopia, 2021 (*n* = 537)

Items	None	Rarely	Sometimes	Frequently	Always
How often are you maintain physical distance?	5 (0.9%)	130 (24.2%)	299 (55.7%)	99 (18.4%)	4 (0.8%)
How often are you avoiding larger gatherings?	11 (2.0%)	181 (33.7%)	267 (49.7%)	76 (14.2%)	2 (0.4%)
How often are you avoiding touching your face, eyes, mouth, and nose?	117 (21.8%)	207 (38.5%)	197 (36.7%)	15 (2.8%)	1 (0.2%)
How often are you washing your hands with water and soap or sanitizers?	19 (3.5%)	204 (38.0%)	230 (42.8%)	73 (13.6%)	11 (2.1%)
How often are you avoiding contact with people who had fever and cough?	12 (2.2%)	219 (40.8%)	221 (41.2%)	77 (14.3%)	8 (1.5%)
How often are you wearing facemasks when you are at work or outside the home?	20 (3.7%)	161 (30.0%)	285 (53.1%)	68 (12.7%)	3 (0.5%)
How often are you avoiding using public transportation?	39 (7.2%)	138 (25.7%)	262 (48.8%)	96 (17.9%)	2 (0.4%)
How often are you avoiding unprotected contacting (touching) of frequently contacted surfaces	21 (3.9%)	259 (48.2%)	211 (39.3%)	44 (8.2%)	2 (0.4%)
How often are you staying home to prevent COVID‐19 infection?	120 (22.3%)	68 (12.7%)	250 (46.6%)	97 (18.0%)	2 (0.4%)

### 3.5 Level of trust and perceived self‐efficacy

Regarding COVID‐19, above half 299 (55.7%) of older adults had high level of trust in government. About one quarter 153 (28.5%) of the older adults had low trust level in medical professionals concerning COVID‐19. The perceived self‐efficacy to practice the prevention methods of COVID‐19 was measured by two items, and the computed mean score was 7.89. About 205 (38.2%) of the older adults had poor perceived self‐efficacy to practice the prevention methods for COVID‐19 **(**Table 5 **).**

**TABLE 5:** Level of trust among older adults in Southern Ethiopia, 2021 (*n* = 537)

Variable	Categories	Frequency	Percent
Level of trust in government	High	299	55.6
	Medium	163	30.4
	Low	75	14.0
Level of trust in science	High	276	51.4
	Medium	180	33.5
	Low	81	15.1
Level of trust in medical professional	High	287	53.4
	Medium	97	18.1
	Low	153	28.5

### 3.6 Disease related conditions

About 24 (4.5%) of the study participants had reported history of COVID‐19. The participants having a previous family history of COVID‐19 accounted for 13 (2.4%). About 51 (9.5%) of participants had a history of chronic disease such as diabetes mellitus, respiratory disease, hypertension, and cardiovascular disease (Table 6).

**TABLE 6:** Disease related condition among older adults in Southern Ethiopia, 2021 (*n* = 537)

Variables	Yes	No
Having previous history of COVID‐19	24 (4.5%)	513 (95.5%)
Having previous COVID‐19 history of family	13 (2.4%)	524 (97.6%)
History of chronic diseases	51 (9.5%)	486 (90.5%)

### 3.7 Factors associated with low risk perception of COVID‐19

Variables significantly associated with low COVID‐19 risk perception level in multivariate logistic regression were age, preventive practice of COVID‐19, trust in medical professionals, perceived self‐efficacy, and history of COVID‐19. Those with an age range of 65 to 74 years were almost five times (AOR = 4.76, 95% CI: 2.35–9.64) more likely to have low risk perception toward COVID‐19 than individuals aged 85 and above. The individuals having poor practice toward preventing COVID‐19 were approximately two times (AOR = 2.39, 95% CI: 1.51–3.78) more likely to have low risk perception than counterparts. Those individuals who have low trust level in medical professionals were two times (AOR = 2.44, 95% CI: 1.45–4.10) more likely to have low risk perception toward COVID‐19 than individuals having high trust level. Individual with having no history of COVID‐19 had more than six times (AOR = 6.45, 95% CI: 2.02–20.58) increased odds of having low risk perception toward COVID‐19 than counterparts (Table 7).

**TABLE 7:** Factors associated with COVID‐19 risk perception level among older adults in Southern Ethiopia, 2021 (*N* = 537)

		COVID‐19 risk perception				
Variables		Low risk perception	High risk perception	COR (95%CI)	*P* value for COR	AOR (95%CI)
Age	65–74	207 (72.1%)	80 (37.9%)	4.74 (2.53–8.90)	<0.001	4.76 (2.35–9.64)^*^
	75–84	53 (26.6%)	146 (73.4%)	0.66(0.35–1.28)	0.22	0.66 (0.32–1.36)
	85 and above	18 (35.3%)	33 (64.7%)	1		1
Knowledge toward coronavirus disease	Poor	36 (69.2%)	16 (30.8%)	2.41 (1.29–4.48)	0.006	1.06 (0.48–2.34)
	Fair	54 (56.2%)	42 (43.8%)	1.37 (0.88–2.16)	0.17	1.36 (0.77–2.42)
	Good	188 (48.3%)	201 (51.7%)	1		1
Preventive practice toward COVID‐19	Poor	189 (65.6%)	99 (34.4%)	3.43 (2.41–4.89)	<0.001	2.39 (1.51–3.78)^*^
	Good	89 (35.7%)	160 (64.3%)	1		1
Trust in government	High	136 (45.5%)	163 (55.5%)	1		1
	Medium	86 (52.8%)	77 (47.2%)	3.53 (2.00–6.23)	<0.001	1.96 (0.99–3.87)
	Low	56 (74.7%)	19 (25.3%)	1.34 (0.91–1.96)	0.14	1.22 (0.75–1.97)

Abbreviations: AOR, adjusted odds ratio; CI, confidence interval; COVID‐19, coronavirus disease 2019; COR, crude odds ratio.

^*^ *p* value less than 0.05.

## 4 DISCUSSION

This study tried to assess the level of risk perception toward COVID‐19 and associated factors among older adults. The proportion of low perception level of risk toward COVID‐19 among older adults was 51.8% with 95% CI between 47.5% and 55.98%. This was higher than a study conducted in South‐West Ethiopia among waiters in the adult age group, which was 46.6%.^12^ However, the finding was lower than studies conducted in Northeast Ethiopia (66.6%)^14^ and Gondar (76.89%).^15^ It was also in contrast to the study conducted in China among college students, which showed the high‐risk perception of COVID‐19.^16^ The study difference may be related with the coronavirus phase difference in which the study was conducted, individual characteristics, and study population. Regarding associated factors, age of the older adults, perceived self‐efficacy to practice the prevention methods; practice toward prevention of coronavirus disease, trust in medical professionals, and previous infection with COVID‐19 was significantly associated with low risk perception level of COVID‐19.

In this study, the oldest old had less likely to have low risk perception toward coronavirus disease compared to the youngest old. This was supported by the evidence that older adults highly perceive the risk of COVID‐19.^17^ The higher the age, the more perceived risk toward COVID‐19.^12^, ^18^, ^19^ In a study conducted in Gondar town, age above 45 years had 41 % increased odds of (AOR = 1.41, 95% CI; 1.19–2.66) perceiving the high risk of COVID‐19.^15^ The perceived risk increased by 4.9% for every one‐year increase of age.^20^ However, the current finding was in contrary to a study conducted in China showing that logistic regression found out that older adults were less likely to worry about infection with COVID‐19.^21^ The difference might be in study location, study period, and data collection tools and procedures.

Individuals with poor self‐efficacy to practice preventive activity of coronavirus were more than three times likely to have low risk perception for COVID‐19. This indicates that personal efficacy had positively related to risk perception, which means having high personal efficacy perceives the risk of COVID‐19 more than those with low personal efficacy.^11^ Moreover, a study conducted in Middle East has indicated that it had a positive relation between self‐efficacy and COVID‐19 risk perception.^22^ Moreover, another study has reported that personal efficacy was directly related to COVID‐19 risk perception.^23^ This shows that conditioning targeted on advancing individual self‐efficacy with preventive practices could reduce the burden of COVID‐19.

Older adults with low trust in medical professionals were high likely to have low risk perception COVID‐19. High trust in the health system adds seeking probability of health workers care in case of the first symptoms of COVID‐19.^23^ Qualitative study has explored that trust in the information sources is one of the factors influencing the perception of the risk of COVID‐19.^24^ Another study has also shown that risk perception was affected by trust in medical professionals.^22^ This might be due to trust in the information forwarded from the medical professionals.

Poorer compliance with preventive measures of COVID‐19 was associated with low perceived risk of coronavirus disease. Risk perception was an independent predictor of protective behaviors related to COVID‐19 (*P* < 0.05) and vice versa.^25^ Another study has shown the positive relation between COVID‐19 risk perception level and practice on prevention of COVID‐19.^12^

Moreover, this study has found that an individual who has not had COVID‐19 had more than six times increased perception of risk of COVID‐19. Moreover, the study has shown that patients with chronic disease (OR = 2.423) had a higher level of risk perceived.^20^ Another study also revealed that the level of risk perception of COVID‐19 was higher for individual acquired chronic disease.^19^ This would bring forth a great understanding of the community's perception of the risk levels of COVID ‐19 and would help to identify a vulnerable individuals for intervention.

Our study had a number of limitations. Mainly, the cross sectional nature of the study was limitation that would make difficult in identifying direction of association. This is also the limitation of our study. The other limitation was not considering the other domains of risk other than psychological dimension of risk perception.

## 5 CONCLUSIONS

In the current study area, the proportion of low risk perception level of COVID‐19 among older adults was 51.8%. The independent associated factors for low perception of risk of COVID‐19 among older adults were age, trust level in medical professionals, preventive practice, perceived self‐efficacy to practice prevention methods, and previous infection with COVID‐19. The findings of this study would help lower income countries to generate evidence‐based policy decisions for older adults during the COVI‐D‐19 pandemic and future pandemic(s). Therefore, to improve the risk perception of COVID‐19 would need advancing the information dissemination through different methods including mass media by medical professionals. It would also need activities targeted on advancing personal self‐efficacy toward preventive practices against COVID‐19. Trust development in information sources would be essential.

## AUTHOR CONTRIBUTIONS

**Tadese Dea Debancho:** Conceptualization; data curation; formal analysis; funding acquisition; investigation; methodology; project administration; resources; software; supervision; validation; visualization. **Eyasu Gambura Gebeyehu:** Conceptualization; data curation; formal analysis; funding acquisition; investigation; methodology; project administration; resources; software; supervision; validation; visualization. **Temesgen Bati Gelgelu:** Conceptualization; data curation; formal analysis; funding acquisition; investigation; methodology; project administration; resources; software; supervision; validation; visualization.

## CONFLICT OF INTEREST

The authors declare no conflict of interest.

## ETHICS STATEMENT

The research protocol was submitted for consideration, review, and approval to the Ethical Review Committee (ERC) of College of Health Sciences and Medicine, Wolaita Sodo University. Then, ethical clearance and approval were obtained from the ERC of the College of Health Sciences and Medicine project number: CHSM/ERC/13/21, Wolaita Sodo University. Written‐informed consent was obtained from each study participant.

### PEER REVIEW

The peer review history for this article is available at https://publons.com/publon/10.1111/irv.13042.

## Data Availability

The authors confirm that all data related to this study are available upon request**.**
